# Aortic Injury Induced by Benzo(a)pyrene and Atherogenic Diet Increased Hepatic FGF21 Expression in C57BL/6J Mice

**DOI:** 10.5812/ijpr-142903

**Published:** 2024-05-11

**Authors:** Farzane Shanehbandpour-Tabari, Fatemeh Gholamnataj, Nahid Neamati, Ebrahim Zabihi, Farideh Feizi, Hadi Parsian

**Affiliations:** 1 Cellular and Molecular Biology Research Center, Health Research Institute, Babol University of Medical Sciences, Babol, Iran; 2 Student Research Committee, Babol University of Medical Sciences, Babol, Iran; 3 Department of Clinical Biochemistry, Babol University of Medical Sciences, Babol, Iran; 4 Department of Toxicology and Pharmacology, Pharmaceutical Sciences Research Center, Babol University of Medical Sciences, Babol, Iran; 5 Cellular and Molecular Biology Research Center, Babol University of Medical Sciences, Babol, Iran; 6 Department of Anatomical Sciences, Faculty of Medicine, Babol University of Medical Sciences, Babol, Iran

**Keywords:** Benzo(a)pyrene, Fibroblast Growth Factor 21, Atherosclerosis

## Abstract

**Background:**

Benzo(a)pyrene (BaP), an environmental toxicant and endocrine disruptor, has been shown to exacerbate atherosclerosis when combined with a high-fat diet. Fibroblast Growth Factor-21 (FGF21), a novel hormone with anti-atherosclerotic properties, is associated with the presence of atherosclerosis and reduces plaque formation in experimental animals.

**Objectives:**

The present study aimed to investigate the chronic effect of BaP injection on hepatic FGF21 expression, as an anti-atherosclerotic hormone, in mice fed with or without an atherogenic diet (AtD).

**Methods:**

Eighteen C57BL/6J male mice (6 weeks) were randomly divided into six groups based on the dosage and diet. Blood samples were collected, and serum cholesterol, triglyceride, HDL-C, LDL-C, and glucose levels were measured. FGF21 expression was assessed by quantitative real-time PCR. Atherosclerotic lesions in mice were studied with Oil Red O (ORO) staining.

**Results:**

Benzo(a)pyrene causes a significant increase in liver FGF21 expression in a dose-dependent manner, and BaP co-exposure with AtD leads to a further increase in FGF21 expression. Additionally, the addition of BaP to AtD significantly increased the serum glucose, cholesterol, and LDL-C levels and accelerated the formation of atherosclerotic lesions. Besides, our findings showed that there is a significant positive correlation between FGF21 expression and glucose, cholesterol, LDL-C, and ORO-positive areas.

**Conclusions:**

Our findings revealed that BaP increases the expression of endogenous FGF21 in treated animals as a compensatory response to protect the heart from atherosclerosis induced by BaP and AtD.

## 1. Background

Polycyclic aromatic hydrocarbons (PAHs) are widespread environmental pollutants found in cigarette smoke, automobile exhaust, and roasted foods. The general population is exposed to PAHs mainly due to contaminated foods, inhalation of polluted air, or cigarette smoking (1). Cigarette smoking is a major source of PAH for the general population (2). Benzo(a)pyrene (BaP), a PAH present in tobacco smoke, has been suggested as the cause of human diseases, including cancers and cardiovascular diseases (3-5). Although it has been shown that BaP causes the progression of atherosclerosis, the underlying mechanism remains unclear. Many studies have focused on how BaP exposure can affect the atherosclerotic process (3, 6-9). Benzo(a)pyrene is a strong aryl hydrocarbon receptor agonist (AhR). The AhR translocates to the nucleus and dimerizes with Aryl hydrocarbon receptor nuclear translocator (ARNT) upon BaP interaction. The AhR-ARNT heterodimer, activated by the agonist, binds to dioxin response elements (DREs) and induces a battery of gene expression (10, 11).

Recently, studies indicate that the activation of the AhR changes hepatic FGF21 expression. The FGF21 promoter contains several putative DREs (12). Fibroblast growth factor 21 (FGF21) belongs to the FGF19 subfamily and is mainly expressed in the liver. It serves as a multifunctional protein that regulates lipid/glucose metabolism and energy balance (13, 14). Fibroblast growth factor 21 deficiency aggravates atherosclerotic plaque formation in the animal model. On the other hand, FGF21 therapy reduces the initiation and progression of atherosclerosis (15). Besides, FGF21 therapy is accompanied by a lipid-lowering effect in non-human primates and reduced atherosclerotic plaque formation in mice (16-18). A recent clinical research revealed that blood levels of FGF21 in smokers were substantially higher than those of non-smokers (19).

## 2. Objectives

In light of the aforementioned, we assumed that the rise in FGF21 levels is likely due to the aromatic compounds, such as BaP, present in the smoke. Therefore, we designed a project to study the effect of BaP, as a potent activator of the AhR, with or without an atherogenic diet, on the expression of FGF21 in the liver of C57BL/6 mice.

## 3. Methods

### 3.1. Experimental Animals

Eighteen male (6-week-old) C57Bl/6J mice weighing 27 ± 4 g were purchased from the Pasteur Institute (Karaj, Iran). The animals were housed in a controlled environment for 1 week before the study (with a normal rodent chow diet and water ad libitum in a room at a mean temperature of 21 - 23 °C). Then, the animals were randomly divided into six groups (3 mice per group): 5 mg/kg/week BaP with an atherogenic diet (5BA), 1 mg/kg/week BaP with an atherogenic diet (1BA), 5 mg/kg/week BaP without an atherogenic diet (5B), 1 mg/kg/week BaP without an atherogenic diet (1B), an atherogenic diet without BaP, and control groups received only corn oil. BaP (purity > 99.8 %, Sigma-Aldrich, St. Louis, MO, USA) was dissolved in corn oil, and intraperitoneal injection was repeated for 16 consecutive weeks. In addition, all injections were given at a volume of 10 mL/kg. The atherogenic diet contained 0.5% cholic acid, 1.25% cholesterol, and 15% fat purchased from the Royan Institute for Animal Biotechnology (RI-AB), Isfahan, Iran.

### 3.2. Biochemical Measurements

After 16 weeks from the beginning of the experiment, peripheral blood was collected under a 12-hour fasting period from the retro-orbital sinus of mice under anesthesia with ketamine (100 mg/kg) / xylazine (10 mg/kg) (Alfasan Co., Netherlands). All blood samples were centrifuged (12000 g for 15 min) and stored at -80 °C. Serum glucose and lipid profile levels, including total cholesterol (TC), low-density lipoprotein-cholesterol (LDL-C), high-density lipoprotein-cholesterol (HDL-C), and triglyceride (TG), were measured using Pars-Azmun enzymatic assay kits (Pars-Azmun co, Iran), and the automated Roche Hitachi 912 (Chemistry Analyzer, Germany).

### 3.3. Quantification of Atherosclerotic Lesion Size

The animals were anesthetized, perfused with phosphate-buffered saline (PBS) (DNA biotech, Tehran, Iran), and fixed with 4% paraformaldehyde (Sigma-Aldrich, St. Louis, MO, USA) via the left ventricle for 15 minutes. Then, all the heart and aorta were removed. The heart was fixed with 4% paraformaldehyde in PBS overnight and transferred into 30% sucrose in PBS solution for 24 hours. In brief, a microtome device (Thermo Fisher Scientific, USA) was used to obtain serial frozen sections (10 μm thickness) until aortic valve cusps were observed. These sections were then stained with Oil Red O (ORO) (Asia pajohesh, Iran) and counterstained with hematoxylin. Tissue sections were evaluated and imaged with a camera-equipped Olympus BX41 microscope (Olympus, Japan; Canon, pc1587. JAPAN). The lesion area size of the 5 sections was averaged for each mouse using Image J software, and the mean lesion size was used for statistical analysis.

### 3.4. En Face Aorta Staining Preparation

Oil Red O staining of the whole aorta, also known as En face ORO staining of the aorta, is considered the gold standard for atherosclerosis research. This technique enables us to survey the entire aorta and identify the location of lipid-rich lesions that occur in endothelial cells (20). To perform En face aorta staining, the entire length of the aorta up to the iliac bifurcation was dissected, and periarterial adipose tissue was removed under a stereomicroscope (Motic SMZ-143 Series, China). The whole aorta was stained with ORO (Sigma-Aldrich, St. Louis, MO, USA) as previously described (21). Subsequently, the whole aorta was surveyed to find ORO-positive areas.

### 3.5. Gene Expression Analysis

Total RNA was extracted from 10 mg of liver tissue using RNXplus (SinaClon, Iran) according to the manufacturer’s protocol and stored at -80 °C until use. RNA concentration was determined using Nanodrop 2000 (Thermo Fisher Scientific, USA), and its integrity was verified by agarose gel electrophoresis. The cDNA synthesis was performed by dissolving 500 ng of total RNA in a mixture of 50 µM random hexamer primer, 4 µL 5x-first strand buffer, 1 µL of 10 mM dNTP, 10 Units of RNase inhibitor, and 100 Units of M-MLV Reverse Transcriptase (Yekta Tajhiz Azma, Iran) to a final volume of 20 µL by adding DEPC-treated water into the mixture. The mixture was incubated at 25 degrees Celsius for 10 minutes, 42 degrees Celsius for 1 hour, and 60 degrees Celsius for 10 minutes. The generated cDNAs were used for real-time PCR testing. Beta-actin was used as a reference gene for the qRT-PCR analysis of FGF 21 gene expression levels. Forward and reverse primer sequences are presented in Table 1. PCR amplification was done using SYBR green PCR master mix according to the manufacturer's protocol, and thermal amplification was as follows: Initial denaturation was done at 95°C for 10 minutes. Three-step gene amplification at 40 cycles (denaturation 15 seconds at 95°C, primer annealing 30 seconds at 62°C and 57°C for Fgf21 and Beta-actin, respectively). The extension step lasted for 45 seconds at 72°C. The melt curve analysis was performed at 83°C. As mentioned above, beta-actin mRNA expression was used as the internal control for each sample, and expression fold change was calculated for each sample based on the comparative CT method (Pfaffl formula). All the experiments were done in duplicate.

**Table 1.** Primers Sequences Used in RT-qPCR

Gene Symbol	Accession No.	Primer Sequence
**Fibroblast growth factor-21 (FGF21)**	NM_020013.4	Forward: 5 '- CTC TCT ATG GAT CGC CTC AC-3 '
		Reverse: 5 ' CAT GGG CTT CAG ACT GGT AC-3 '
**Beta-actin**	NM_007393.5	Forward: 5 '- CAG CCT TCC TTC TTG GGT ATG-3'
		Reverse: 5'- TTG GCA TAG AGG TCT TTA CGG-3'

### 3.6. Statistical Analysis

SPSS software (version 26) was used for the analysis of the data. Differences between several groups were evaluated with the Kruskal-Wallis test, and the Mann-Whitney U test was used to compare the changes of gene expression in mouse liver and for the comparison of biochemical parameter changes between groups. Spearman’s correlation coefficient test was used to investigate the correlation between changes in FGF21 expression and analytical variables. Data are expressed as mean ± standard error of the mean (SEM), and P-values < 0.05 were considered statistically significant.

## 4. Results

### 4.1. Benzo(a)pyrene Exacerbates Atherosclerotic Lesions in Animals Fed an Atherogenic Diet

**Figure 1.**
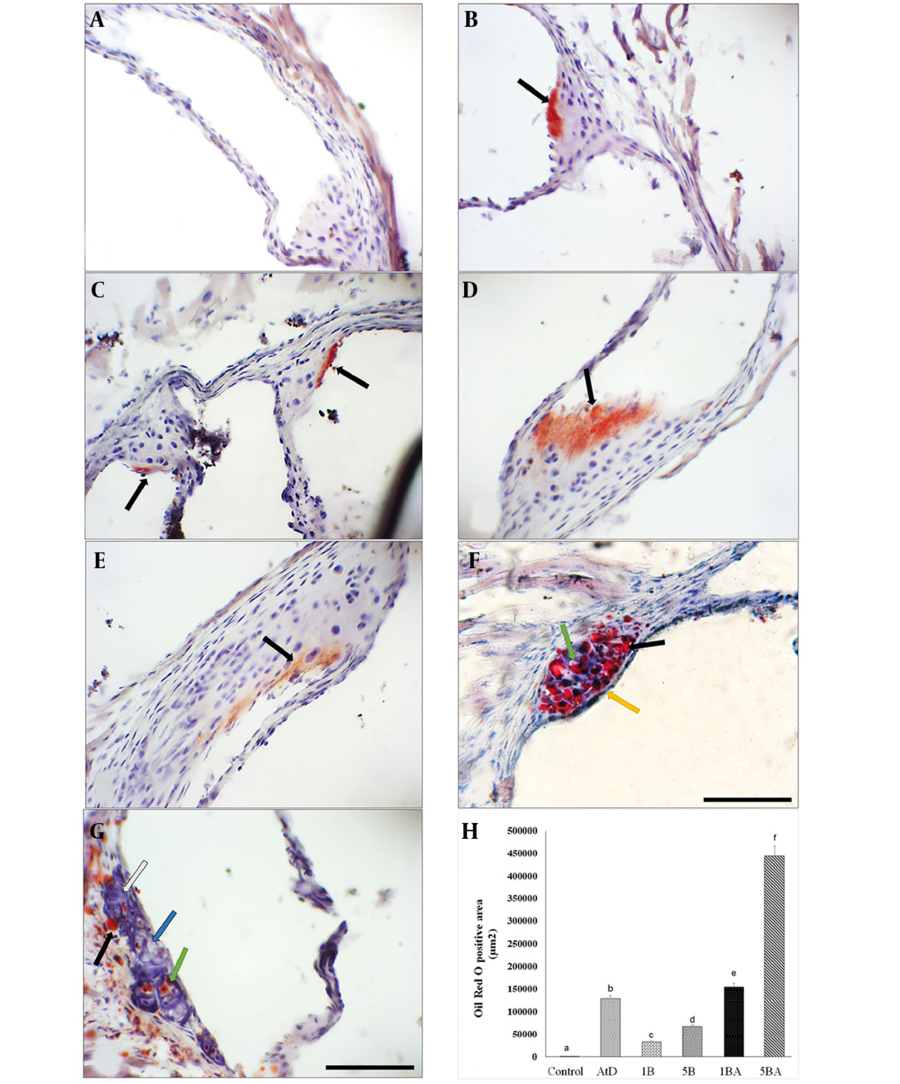
Benzo(a)pyrene (BaP) enhances atherosclerotic plaques in animals fed an atherogenic diet. Microscopic cross-sections (10 µm) of the aortic root were stained with Oil Red O (ORO) (counterstained with hematoxylin) to reveal lipid deposition. Lipid deposition was identiﬁed by its red color. B-E, black arrows indicate fatty streak; F-G, foam cells in the aortic root (green arrow), free lipid droplets in variable sizes (black arrow), intact fibrous cap (yellow arrow), calcification (white arrow), and necrotic core (blue arrow). Scale bar, 100 µm, Original magnification, 40x. H, The ORO positive area among different groups (Kruskal Wallis test, P-value = 0.007) was measured in square micrometers. Different superscript letters (a, b, c, etc.) indicate statistically significant differences at a P-value < 0.05 between the groups. Values represent the average of 3 mice. Error bars indicate S.E. A, control; B, atherogenic diet (AtD); C, 1B (1mg/kg BaP); D, 1BA (AtD + 1mg/kg BaP); E, 5B (5mg/kg BaP); F and G, 5BA (AtD + 5mg/kg BaP)

Following 16 weeks of the experiment, no lesions were observed in the control mice. However, mice that were fed an atherogenic diet or treated with BaP alone exhibited early small fatty streak lesions of atherosclerosis limited to the aortic root without detectable plaque (Figure 1A-E). Atherosclerotic lesions, predominantly composed of fatty streak lesions infiltrated by foam cells, free lipid droplets, a fibrous cap, and a calcified necrotic core (NC), were observed in the aortic root of the 5BA groups (Figure 1F-G). Oil Red O Positive area measurements in groups that were treated with BaP and an atherogenic diet showed significantly higher plaque compared with animals fed a control diet. The ORO-positive area in the aortic root was significantly wider in the 1 mg/kg BaP plus atherogenic diet than in 1mg/kg BaP treated mice (P < 0.05). Measurement of the ORO-positive area revealed that animals treated with 5 mg/kg BaP plus atherogenic diet had considerably greater plaque load than mice treated with 5 mg/kg BaP or atherogenic diet alone (P < 0.05) (Figure 1H). 

### 4.2. En Face Aorta Staining Results

**Figure 2.**
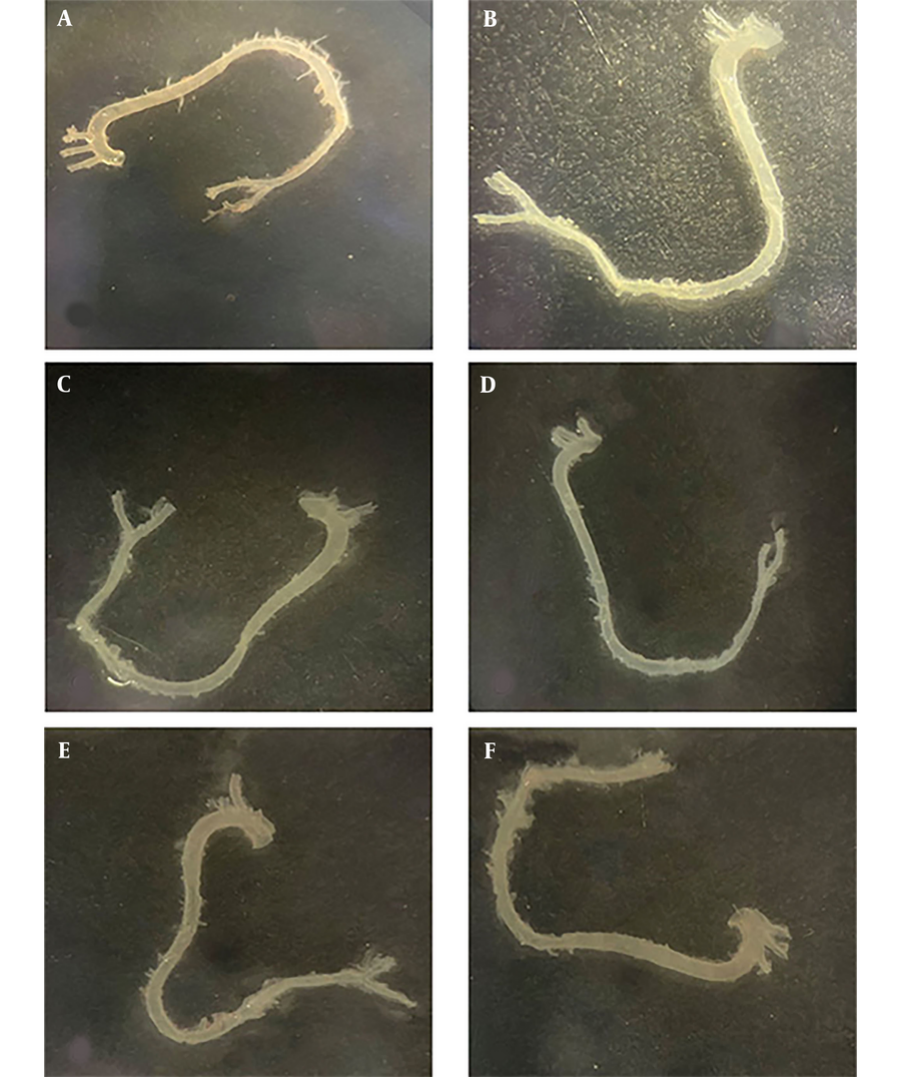
En face view of aortic surface lesions. Oil Red O (ORO) staining was performed on an aorta, and no atherosclerotic lesions were observed in all groups. A, control; B, atherogenic diet (AtD); C, 1B (1mg/kg BaP); D, 1BA (AtD + 1mg/kg BaP); E, 5B (5mg/kg BaP); F, 5BA (AtD + 5mg/kg BaP)

The En face ORO staining method was performed to trace atherosclerotic lesions throughout the entire length of the aorta, from the ascending to the iliac aortic regions. Based on the data obtained from the En face staining results, no lesions were detected in mice fed an atherogenic diet or in BaP-treated mice (with or without an atherogenic diet) (Figure 2). 

### 4.3. Effect of Benzo(a)pyrene on Biochemical Parameters

**Figure 3.**
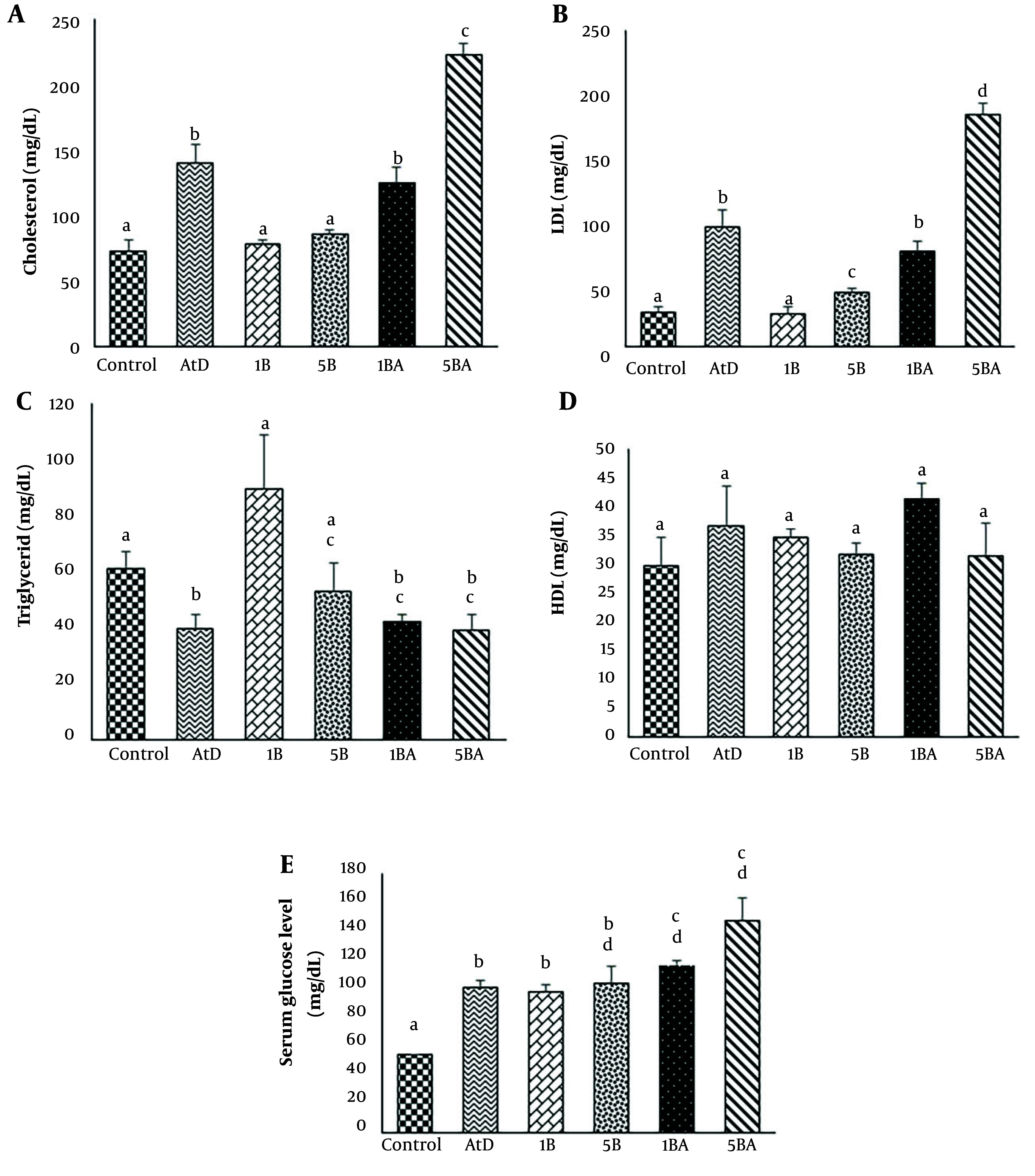
Serum lipid profiles and glucose levels in all experimental groups. Serum lipid values are given as mg/dL and were shown as the average of 3 mice. Different superscript letters (a, b, c, etc.) indicate statistically significant differences at a P-value < 0.05 between the groups. Error bars indicate standard error (S.E) [Abbreviation: LDL, low-density lipoprotein; HDL, high-density lipoprotein; AtD, levels, atherogenic diet; 1B, (1 mg/kg BaP); 1BA, (AtD + 1 mg/kg BaP); 5B, (5 mg/kg BaP); 5BA, (AtD + 5 mg/kg BaP)].

The serum concentrations of TC and LDL-C in the group treated with an atherogenic diet were significantly higher than the control group (vehicle) (P < 0.05). TC and LDL-C were increased in the animals treated with BaP (1 and 5 mg/kg) plus an atherogenic diet compared to just BaP-treated animals. Although TC and LDL-C levels were eventually doubled in the BaP (1 or 5 mg/kg) plus an atherogenic diet, triglyceride concentration was reduced by approximately 50% in the treated groups. Moreover, HDL-C levels did not show a significant difference in the atherogenic diet with and without the BaP group. Benzo(a)pyrene treatment with or without an atherogenic diet resulted in a significant increase in blood glucose levels. All treatment groups had significantly higher serum blood glucose levels than the vehicle-control group (P < 0.05). Additionally, compared with groups that received BaP with an atherogenic diet, serum glucose levels were lower in all groups that received BaP alone. In terms of blood glucose levels, there was no difference between groups in doses 1 and 5 mg/kg BaP. However, in the 1 and 5 mg/kg BaP plus atherogenic diet groups (in both groups), the serum glucose levels compared with the atherogenic group showed a significant increase (P-value < 0.05) (Figure 3 and Table 2). 

**Table 2.** Serum Lipid and Glucose Values are Given as mg/dL and Values Represent the Average of 3 Mice ^a^

Groups Variables	Control	AtD	1B	5B	1BA	5BA	P-Value ^b^
**TC**	72.4 ± 8.2 ^A^	139.3 ± 15.1 ^B^	78.0 ± 2.6 ^A, C^	85.6 ± 2.6 ^A, C^	125 ± 11.2 ^B^	222.6 ± 8.8 ^D^	0.01
**LDL-c**	27.1 ± 4.7 ^A^	94.7 ± 13.0 ^B^	26.4 ± 5.1 ^A^	43.2 ± 2.9 ^C^	75.0 ± 8.7 ^B^	183.4 ± 9.0 ^D^	0.007
**TG**	61.0 ± 6.2 ^A^	39.6 ± 5.4 ^B^	89.6 ± 19.6 ^A^	53.3 ± 10.1 ^A, C^	42.6 ± 2.3 ^B, C^	39.3 ± 5.8 ^B, C^	0.05
**HDL-c**	29.6 ± 5.0 ^A^	36.6 ± 6.9 ^A^	34.6 ± 1.4 ^A^	31.7 ± 1.8 ^A^	41.3 ± 2.7 ^A^	31.3 ± 5.8 ^A^	0.43
**Glucose**	54.0 ± 0.5 ^A^	101.0 ± 4.9 ^B^	98 ± 4.3 ^B^	104.0 ± 11.8 ^B, D^	116.3 ± 3.1 ^C, D^	147.6 ± 15.8 ^C, D^	0.02

Abbreviation: LDL, low-density lipoprotein level; HDL, high-density lipoprotein level; AtD, atherogenic diet; 1BA, 1B (1 mg/kg BaP) (AtD + 1 mg/kg BaP); 5BA, 5B (5 mg/kg BaP) (AtD + 5 mg/kg BaP).

^a^ Different superscript capital letters in the table indicate statistically significant differences at a P-value < 0.05 between the groups.

^b^ Kruskal–Wallis test P-values for the different groups are indicated in the table.

### 4.4. Benzo(a)pyrene Treatment Increase Hepatic Fibroblast Growth Factor-21 Expression Induced by Atherogenic Diet

**Figure 4.**
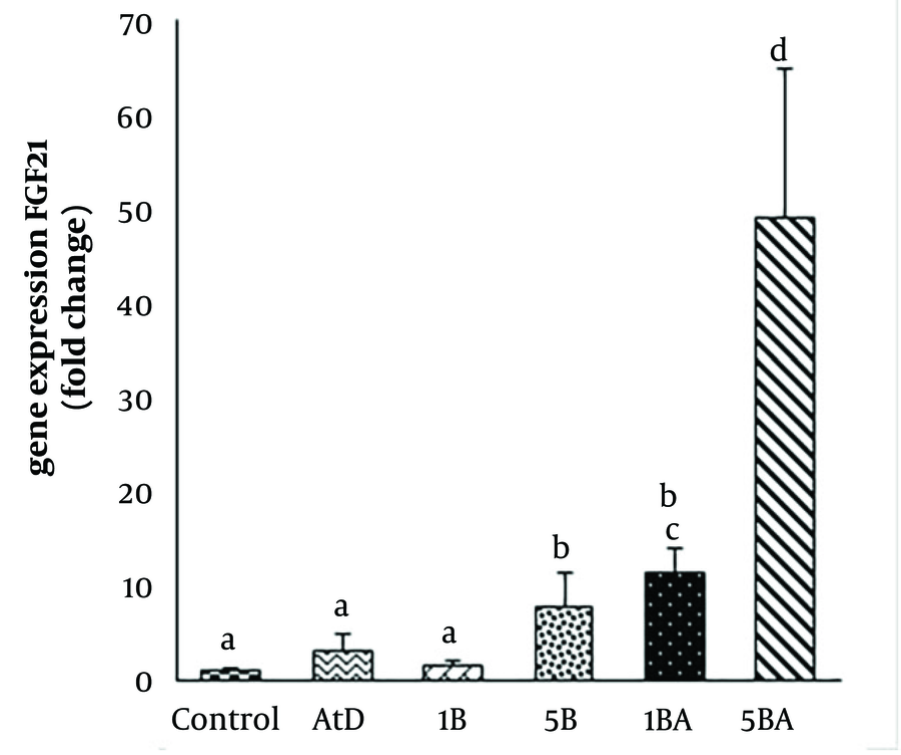
Hepatic fibroblast growth factor-21 (FGF21) expression levels among the different groups (Kruskal Wallis test, P-value = 0.02). Different superscript letters (a, b, c, etc.) indicate statistically significant differences at a P-value < 0.05 between the groups [Abbreviations: AtD, control atherogenic diet; 1B, (1mg/kg BaP); 1BH, (AtD + 1mg/kg BaP); 5B, (5mg/kg BaP); 5BH, (AtD + 5mg/kg BaP)]

**Figure 5.**
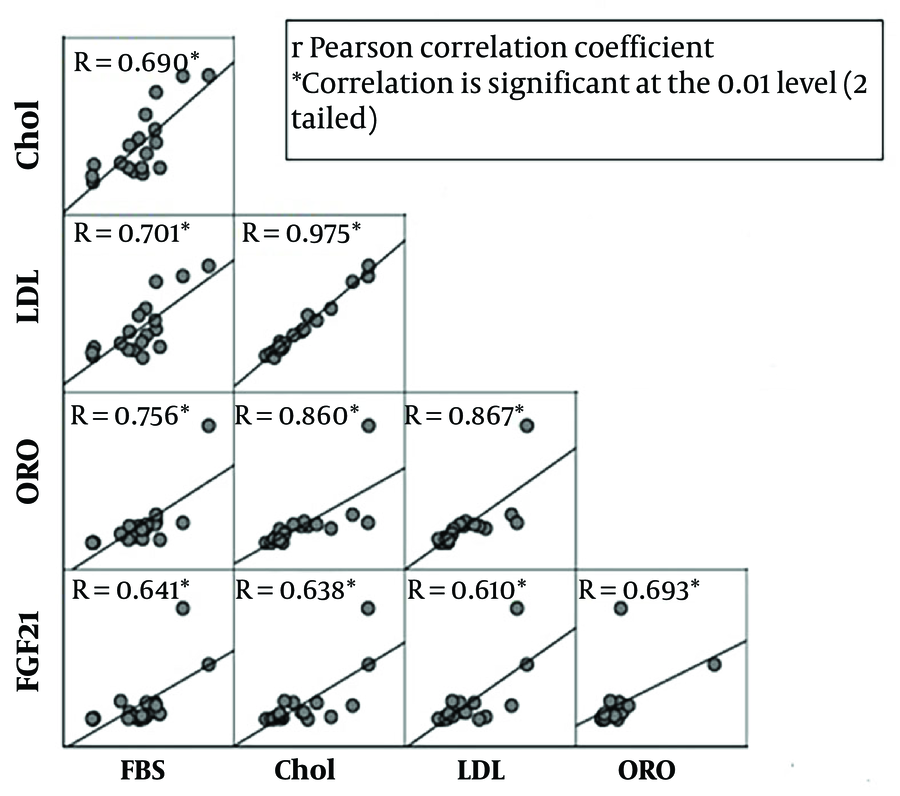
Correlation analysis between hepatic Fibroblast growth factor-21 (FGF21) and serum cholesterol, LDL, glucose levels, and ORO (Abbreviations: TC, total Cholesterol; LDL-C, low-density lipoprotein; HDL-C, high-density lipoprotein; and ORO, ORO positive area)

To determine whether FGF21 expression is changed in hepatocytes during treatment with BaP and an atherogenic diet, the expression of FGF21 in the liver was analyzed. Our results indicated that the expression level of FGF21 was increased in BaP-exposed mice in a dose- and diet-dependent manner. In the group that received only an atherogenic diet, the FGF21 expression level was found to be three times higher. At the lowest dosage of BaP (1 mg/kg), the expression level of FGF21 was increased 1.5 times more than the control group, while exposure to the highest dose (5 mg/kg) significantly increased the expression level of FGF21 with a fold-change of 7.8 (P-value < 0.05) compared with the control. Furthermore, the hepatic FGF21 expression was 11-fold higher in the 1 mg/kg BaP plus atherogenic diet group and 48.8-fold higher in the atherogenic diet plus 5 mg/kg BaP group compared with the control group, respectively (P-value < 0.05) (Figure 4). There was a significant correlation between FGF21 expression and serum levels of LDL-C, TC, and glucose (R = 0.610, 0.638, and 0.641; P-value = 0.007, 0.004, 0.004, respectively). In addition, a significant correlation between FGF21 and ORO positive area (R = 0.69; P-value = 0.001) was observed (Figure 5). 

## 5. Discussion

Exposure to BaP, a PAH, has been suggested as a risk factor for the induction and progression of atherosclerosis (22-24). This study aimed to determine the effect of BaP on FGF21 expression levels. Therefore, we investigated the combined effects of BaP and an atherogenic diet on the expression of FGF21 in the liver of C57bl/6 mice. Our results demonstrated that BaP increases FGF21 expression levels in the liver, and mice treated with both BaP and an atherogenic diet showed an enhanced rise in FGF21 expression. Therefore, chronic consumption of an atherogenic diet augmented BaP-induced FGF21 expression. A recent study supported that mice treated with an atherogenic diet had increased expression of FGF21 and FGFR1 in the aorta as well as elevated serum levels of FGF21 in mice (25). Moreover, serum FGF21 levels have also increased in patients with cardiovascular diseases. In animal studies, FGF21 therapy reduces atherosclerotic plaques (26, 27). Therefore, increased endogenous FGF21 expression is proposed as a compensatory reaction and a defensive mechanism in response to vascular injury in mice after prolonged exposure to BaP and an atherogenic diet.

Serum glucose levels were significantly higher in all experimental groups compared to the control group. A similar study showed that chronic exposure to BaP can increase the risk of type 2 diabetes by inducing pro-inflammatory cytokines such as IL-1beta and TNF-alpha (28). Additionally, we found that mice co-treated with the atherogenic diet and BaP had higher serum glucose levels than those treated with BaP alone. Consistent with our results, they indicated that adding BaP to the high-fat diet significantly reduced the expression of incretin peptide-1 glucagon-like peptide (which has an essential role in insulin secretion) and increased glucose levels. Therefore, to better understand the underlying mechanisms of the induction of high serum glucose levels, activation of inflammatory pathways, or reduction of incretin peptide-1 glucagon-like peptide expression, further studies are needed.

The concentrations of TG were lower in BaP-treated mice with or without an atherogenic diet. This result is consistent with a recent study that indicates FGF21 reduces plasma triglycerides by accelerating fatty acid catabolism in the liver and adipose tissue (29). Since higher FGF21 mRNA expression was observed in mice that received BaP with an atherogenic diet, a lower serum triglyceride concentration can be correlated with FGF21 expression. Moreover, the formation of atherosclerotic plaques was observed in animals whose serum concentrations of atherogenic lipids were elevated. Our results indicated that the combined exposure to BaP and an atherogenic diet further increased TC and LDL concentrations. Furthermore, more fat deposition was observed in the aortic roots of mice exposed to high-dose BaP plus an atherogenic diet compared with groups treated only with an atherogenic diet. Additionally, BaP-treated animals showed early atherosclerosis lesions (fatty streaks) like AtD-treated animals, unlike Curfs et al.'s finding that reported BaP does not initiate atherosclerosis lesions alone (24). Therefore, it is suggested that BaP can initiate atherosclerosis, and co-exposure with an atherogenic diet exacerbates the severity of lesions and consequently increases FGF21 expression. Since FGF21 expression levels correlate with serum concentrations of LDL and TC and lesion size and intensity, we can assume that FGF21 expression increases in response to high lipid concentration and atherogenic lesions. Recent studies have shown that FGF21 expression in the heart increases during cardiac stress to mediate compensatory stress responses (30, 31). Furthermore, the expression of endogenous FGF21 increases in vascular calcification, and exogenous FGF21 has been shown to ameliorate aortic calcification (32). Therefore, this rise in FGF21 level may be a compensatory response to protect the heart from atherosclerosis induced by BaP and an atherogenic diet. On the other hand, another study indicated that monkeys treated with a high-fat diet showed FGF21 resistance due to down-regulation of FGF21 co-receptor β-klotho in the white adipose tissue (33). Besides, Fisher et al. reported that feeding mice with a high-fat/high-sucrose diet resulted in FGF21 resistance in the liver and adipose tissues (34). We could not analyze the status of serum FGF21 protein and beta-Klotho in the aorta to assess FGF21 resistance. Therefore, whether elevated FGF21 expression is due to FGF21 resistance or induced to protect the heart from atherosclerosis caused by an atherogenic diet and chronic BaP exposure needs further investigation.

### 5.1. Conclusions

In summary, our study indicates that chronic exposure to BaP increases the concentration of atherogenic lipids and causes aortic wall injury in C57BL/6 J mice. Additionally, the combined intake of AtD and BaP exacerbates the aforementioned injuries caused by BaP and consequently significantly increases FGF21 expression. Notably, endogenous FGF21 expression significantly increases in atherosclerosis disease, so this increase is a compensatory response to aortic damage induced by BaP and AtD. Surely, highlighting the relationship between FGF21 and BaP will open a window for further understanding of the mechanism of BaP in cardiovascular disease and atherosclerosis.

## Data Availability

The data presented in this study are uploaded during submission as a supplementary file and are openly available for readers upon request.
